# Non-Meckel Ileal Diverticulum Incarcerated Within a Strangulated Inguinal Hernia: A Case Report

**DOI:** 10.7759/cureus.75509

**Published:** 2024-12-10

**Authors:** Baraa Chkir, Ammara Salam, Shua Haq, Moustafa Mansour

**Affiliations:** 1 Urology, Royal Albert Edward Infirmary, Wigan, GBR; 2 General Surgery, North Manchester General Hospital, Manchester, GBR; 3 Colorectal Surgery, North Manchester General Hospital, Manchester, GBR; 4 Upper Gastrointestinal Surgery, North Manchester General Hospital, Manchester, GBR

**Keywords:** ileal diverticulum, inguinal hernia, laparoscopic repair, non-meckel diverticulum, strangulated hernia

## Abstract

Non-Meckel small bowel diverticula, particularly ileal diverticula, are rare, especially when incarcerated within an inguinal hernia sac. This case involves an 80-year-old man who presented with a newly noticed tender, irreducible lump in his left groin, accompanied by symptoms of bowel obstruction such as inability to pass flatus and vomiting. His medical history included a previous right inguinal hernia repair. Physical examination and laboratory tests indicated a strangulated hernia, which was confirmed by a contrast-enhanced computed tomography scan showing small bowel obstruction at the neck of the left inguinal hernia. The patient underwent a laparoscopic mesh repair, during which a non-Meckel ileal diverticulum was discovered within the hernia sac alongside a bruised but viable segment of the small bowel. The incarcerated diverticulum was gently reduced, and the hernia was successfully repaired using a mesh. The patient had an uneventful recovery and was discharged in a stable condition. This case highlights the importance of considering rare causes of small bowel obstruction in elderly patients presenting with hernias. Prompt imaging and surgical intervention are crucial to prevent serious complications such as bowel ischemia and perforation. The successful laparoscopic approach demonstrated minimal invasiveness and facilitated a swift postoperative recovery, underscoring its effectiveness in managing such uncommon clinical scenarios.

## Introduction

Non-Meckel small bowel diverticula (NMSBD) are rare entities, with an incidence ranging between 0.3% and 1.3% in various autopsy studies [1]. Among the different types of NMSBD, jejunal and ileal diverticula are particularly uncommon, and have been reported in 1% to 2% of the general population. They predominantly affect elderly individuals with a male-to-female ratio of 2:1 [2,3]. Unlike Meckel's diverticulum, which is a true congenital diverticulum involving all the layers of the bowel wall, NMSBD are typically acquired pseudodiverticula resulting from high intraluminal pressure or weakening of the intestinal wall [2,4,5]. These diverticula are characterized by the protrusion of the mucosal or submucosal layers through the muscularis propria, and are often associated with conditions such as motility disorders or connective tissue weaknesses [2,4,5].

Jejunal and ileal diverticula are generally asymptomatic and often discovered incidentally during imaging or surgical procedures [2]. However, when complications such as bleeding, perforation, inflammation, or less frequently, strangulation occur, these diverticula can mimic more commonly encountered acute abdominal conditions [6,7]. On the other hand, strangulated hernias are a well-known cause of bowel ischemia and obstruction, requiring immediate surgery [8,9]. The incarceration of small bowel diverticula within a hernia sac, especially an inguinal hernia, is exceedingly rare, and significant diagnostic difficulties arise due to the non-specific clinical symptoms. The entrapped incarcerated diverticulum may become ischemic and lead to strangulation, and if not promptly managed, may progress to perforation, compounding the patient's morbidity and mortality risks [7,10].

This case report highlights a rare presentation of a non-Meckel ileal diverticulum within a strangulated inguinal hernia. By detailing the clinical presentation, diagnostic challenges, and surgical management strategies, this report aims to contribute to the limited literature on this infrequent yet clinically significant condition.

## Case presentation

An 80-year-old man presented to North Manchester General Hospital with a newly noticed lump in his left groin, first observed on the morning of admission. He reported an inability to pass flatus and had experienced two episodes of vomiting. His medical history included a transient ischemic attack, atrial fibrillation, and asthma. He had previously undergone an open mesh repair of a right inguinal hernia. The patient lived alone and had limited mobility. He was able to walk up to ten houses at best. He required assistance with shopping and some activities of daily living.

On physical examination, a scar from the previous right inguinal hernia repair was noted. The abdomen was soft and non-tender. A tender, irreducible left inguinal hernia was palpated without associated skin changes. There was no cough impulse. Laboratory investigations (outlined in Table 1) revealed mild anemia, leucocytosis, and raised lactate. The National Emergency Laparotomy Audit (NELA) score was calculated to be 6.88%, indicating a high risk.

**Table 1:** Initial laboratory results

Lab test	Patient's values	Normal values
Hemoglobin (g/L)	128	130-180
Platelets (x 10^9^/L)	215	150-450
White Blood Cells count (x 10^9^/L)	13.9	4.0-11.0
Lactate (Venous blood gas; mmol/L)	2.7	<1

**Figure 1:**
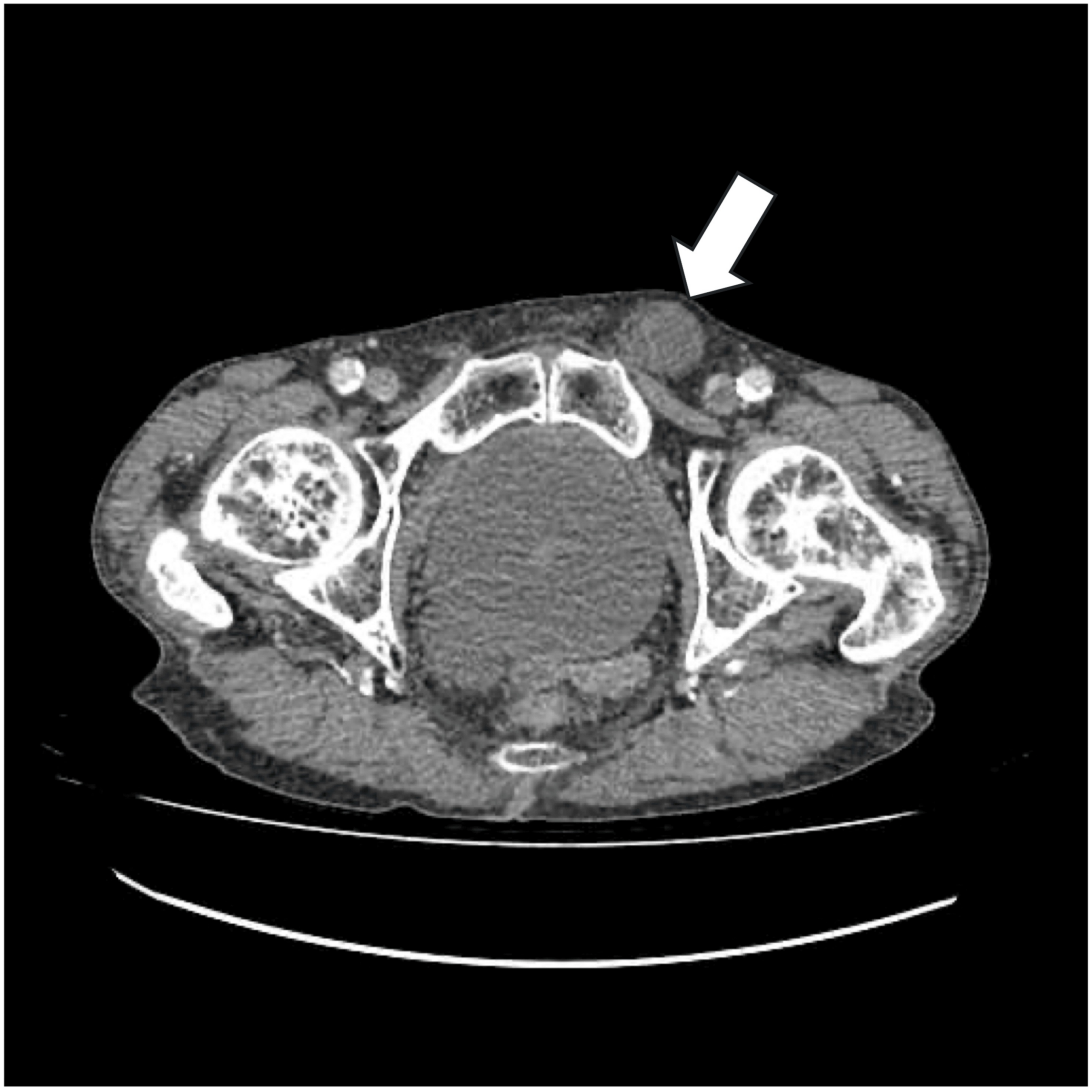
CT abdomen and pelvis showing strangulated left inguinal hernia (white arrow)

A contrast-enhanced computed tomography (CT) scan of the abdomen and pelvis (Figure 1) demonstrated multiple dilated, fluid-filled loops of the small bowel with a transition point at the narrow neck of a left-sided inguinal hernia, which appeared pinched. The hernia sac contained fluid, and the small bowel loops downstream had collapsed, consistent with an acute mechanical small bowel obstruction with strangulation at the hernia neck.

**Figure 2:**
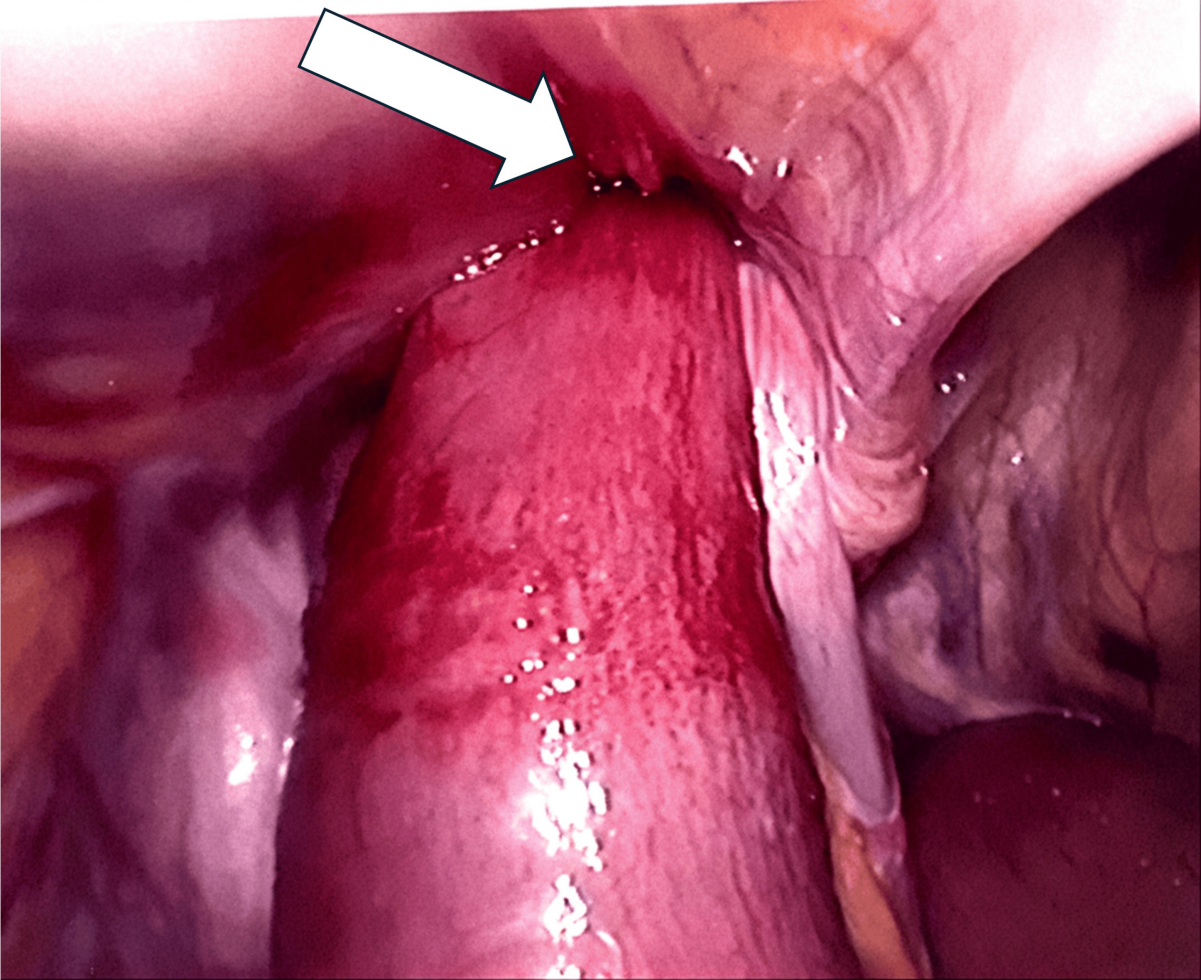
Loop of the small bowel entering the hernia orifice (white arrow)

**Figure 3:**
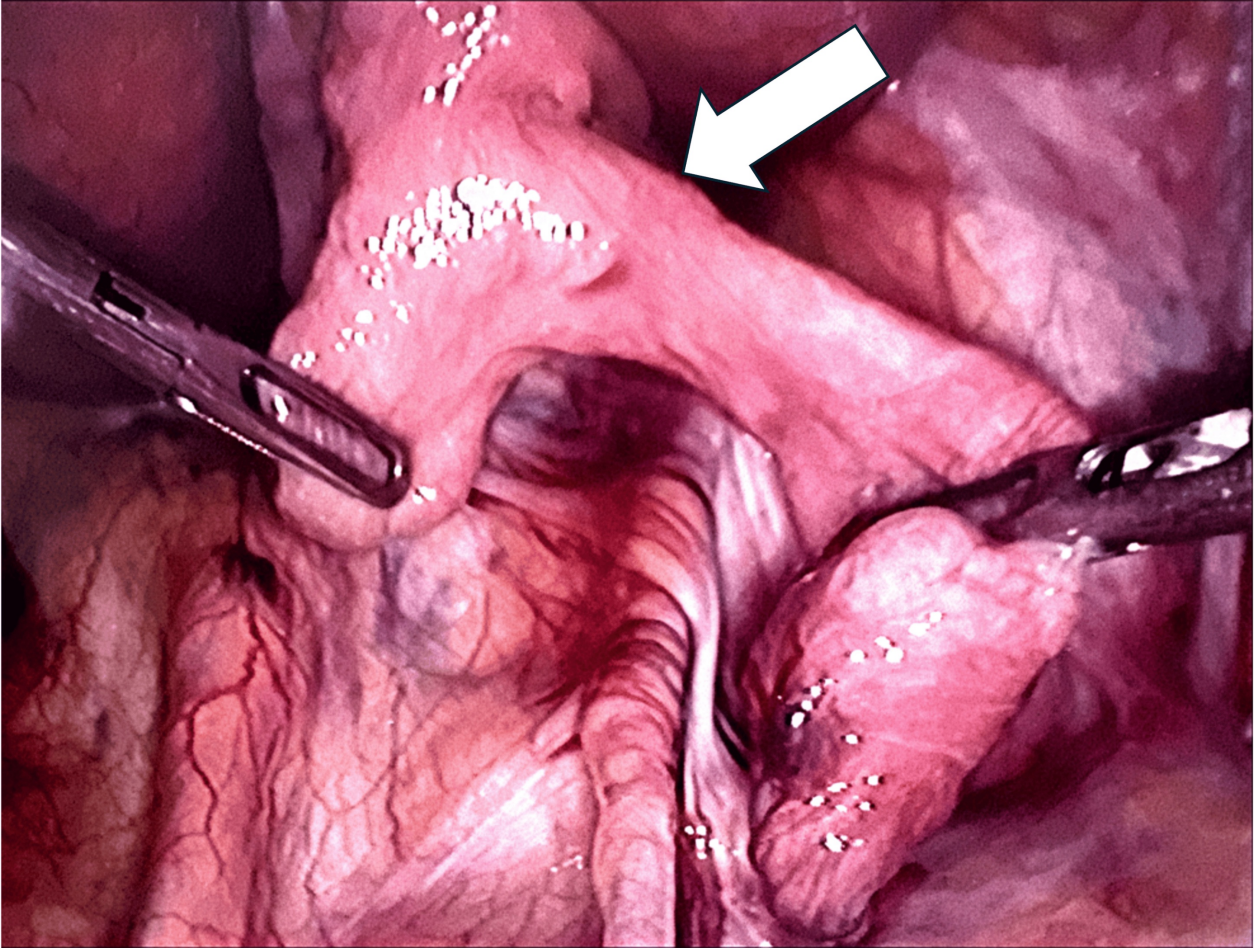
Ileal diverticulum (white arrow) found incarcerated in the hernia sac

**Figure 4:**
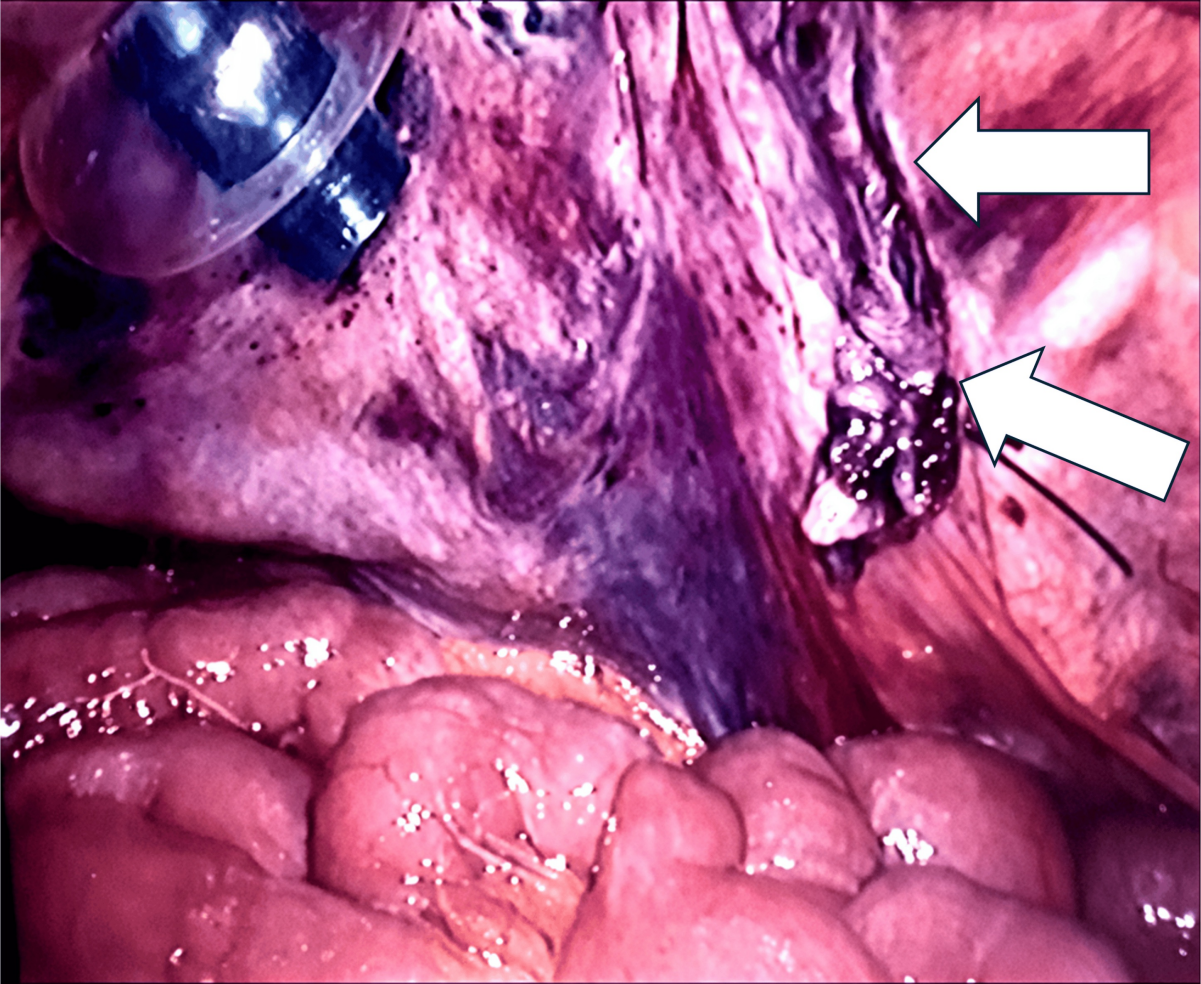
Post mesh repair with peritoneal flap covering the mesh (white arrows)

The patient consented for a laparoscopic mesh repair of the left inguinal hernia. Intra-operatively (Figures 2-4), a direct hernia sac containing a loop of mid-small bowel was identified. The bowel appeared bruised but viable, with preserved peristalsis. Additionally, a healthy segment of small bowel with an ileal diverticulum was found incarcerated within the sac. An indirect hernia sac was also present. The small bowel was gently retracted from the hernia sac, and both the direct and indirect hernia sacs were reduced. A 15 × 15 cm mesh was placed and secured with absorbable tackers medially and laterally, covered by an overlapping peritoneal flap. The bruised segment of small bowel was reassessed for viability and peristalsis. A Robinson drain was placed in the pelvis and secured.

Post-operatively, the patient had an uneventful recovery and was discharged in a stable condition.

## Discussion

Non-Meckel small bowel diverticulosis predominantly affects elderly individuals and is often associated with comorbid conditions such as connective tissue disorders, intestinal dyskinesia, or chronic constipation [1,4]. The pathogenesis is believed to be related to dyskinesia of the smooth muscle and myenteric plexus abnormalities, leading to increased intraluminal pressures and subsequent herniation of the mucosal and submucosal layers through weak points in the muscularis propria [5].

The clinical presentation of NMSBD are highly variable, ranging from asymptomatic incidental findings to acute abdomen due to complications such as diverticulitis, bleeding, perforation, or obstruction [3,6]. Incarceration of a small bowel diverticulum within an inguinal hernia sac is an exceedingly rare event with very few cases reported in the literature [7,10]. This rarity contributes to the diagnostic challenge, as the symptoms often mimic the more common causes of small bowel obstruction.

In our case, the patient's non-specific symptoms of vomiting and inability to pass flatus, combined with a tender, irreducible inguinal hernia, raised the suspicion of a strangulated hernia causing small bowel obstruction. Laboratory findings, including leucocytosis and elevated lactate levels, suggested a possible ischemic process due to compromised bowel circulation.

Contrast-enhanced CT scanning is the imaging modality of choice for evaluating suspected small bowel obstruction. It provides critical information regarding the site and cause of obstruction, as well as signs of bowel ischemia or strangulation [1,11]. However, CT imaging may not always detect small bowel diverticula, especially when complicated by incarceration [11]. In this patient, the CT scan revealed features consistent with an acute mechanical small bowel obstruction due to strangulation at the level of the inguinal hernia neck, but did not specifically identify the incarcerated diverticulum.

Surgical intervention is imperative in cases of strangulated hernias to prevent further complications such as bowel necrosis and perforation [9]. The choice between open and laparoscopic repair depends on various factors, including the patient's clinical status and the surgeon's expertise. Laparoscopic repair offers advantages such as reduced postoperative pain, shorter hospital stays, and quicker return to normal activities [12]. In this case, the laparoscopic approach facilitated thorough exploration and effective management of both the direct and indirect hernia sacs.

Intra-operatively, careful assessment of bowel viability is crucial. In cases where the incarcerated diverticulum is viable, as in our patient, reduction and hernia repair without bowel resection may suffice [5]. If the diverticulum is inflamed or necrotic, segmental resection with primary anastomosis is recommended to prevent postoperative complications [5,7]. The decision to use a mesh in hernia repair must be made cautiously. In the absence of contamination or bowel perforation, mesh repair is acceptable and associated with lower recurrence rates [13].

Postoperative outcomes in patients with complications of NMSBD are generally favorable when prompt surgical intervention is undertaken [7]. Our patient had an uneventful recovery and was discharged in a stable condition. Regular follow-up is essential to monitor for potential complications or recurrence.

This case highlights the importance of considering rare causes of small bowel obstruction in elderly patients presenting with strangulated hernias. Awareness of such atypical presentations can facilitate prompt diagnosis and appropriate management, ultimately improving patient outcomes. Given the limited number of reported cases, further documentation and study are necessary to enhance understanding and guide clinical practice.

## Conclusions

This case report highlights a rare occurrence of a non-Meckel ileal diverticulum incarcerated within a strangulated inguinal hernia. It emphasizes the need for clinicians to maintain a high index of suspicion for small bowel diverticula as a potential cause of small bowel obstruction in elderly patients with hernias. Early diagnosis through appropriate imaging and prompt surgical intervention are paramount in reducing morbidity and mortality associated with this condition. In this case, laparoscopic mesh repair proved to be an effective and safe surgical approach, facilitating a favorable postoperative outcome.
